# Supplementary material for: TNP Analogues Inhibit the Virulence Promoting IP3-4 Kinase Arg1 in the Fungal Pathogen Cryptococcus neoformans
Source: Biomolecules. 2022 Oct 20;12(10):1526. doi: 10.3390/biom12101526 (PMC9599641; doi:10.3390/biom12101526)
Supplement: Supplementary file 1 [file biomolecules-12-01526-s001.zip › biomolecules-1963318-supplementary.pdf]

General procedure I for the first nucleophilic aromatic substitution in the 6-position (2a and 2b) (Scheme 1)

To a solution of 6-chloro-2-fluoropurine (1 mmol, 1.00 equiv.) in *n*-butanol (4 mL), was added *N*,*N*-diisopropylethylamine (1.41 equiv.). The mixture was stirred at room temperature for 5 minutes. To the mixture, was added an amine (1.02 equiv.). The mixture was warmed to 65 °C and stirred for 4 hours. Then the reaction mixture was concentrated *in vacuo* and to the residue, was added cold water. The precipitate was filtered, washed with cold water and purified.

General procedure II for the second nucleophilic aromatic substitution in the 2-position (9 to 29 (excluding 14 and 17), 32, 35, 38 and 39) (Scheme 1)

To a solution of a *N*6-substituted-2-fluoro-9*H*-purin-6-amine (1 mmol, 1.00 equiv.) in *n*-butanol (4 ml), was added *N*,*N*-diisopropylethylamine (2.20 equiv.). The mixture was stirred at room temperature for 5 minutes. To the mixture, was added an amine (2.00 equiv.). The mixture was heated to reflux and stirred overnight (16 hours). Then the reaction mixture was concentrated *in vacuo* and purified.

General procedure III for the reductive amination in the 2-position (5a to 5c) (Scheme 2)

To a solution of 2-amino-6-chloropurine (1 mmol, 1.00 equiv.) in ethyl acetate (4 mL), was added a benzaldehyde (1.20 equiv.). The mixture was cooled to 0 °C and was added trifluoroacetic acid (2.24 equiv.) and sodium triacetoxyborohydride (1.50 equiv.). The reaction was warmed to room temperature and stirred overnight (16 hours). The mixture was quenched with 10% aq. NaOH solution (20 mL) to pH ~ 8-9 and extracted with ethyl acetate (3 x 20 mL). The organic extracts were combined, washed with brine (50 mL) and dried over $MgSO_4$. Then it was concentrated *in vacuo* and purified.

General procedure IV for the nucleophilic aromatic substitution in the 6-position (14, 30, 31, 36 and 41 to 44) (Scheme 2)

To a solution of *N*2-substituted-6-chloro-9*H*-purin-2-amine (1 mmol, 1.00 equiv.) in *n*-butanol (4 mL), was added an amine (1.50 equiv.) and triethylamine (1.00 equiv.). The reaction was heated to reflux and stirred for 5 hours. Then the reaction mixture was concentrated *in vacuo* and purified.

N-Benzyl-2-fluoro-9*H*-purin-6-amine (2a) [39]

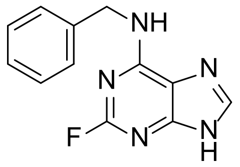

General procedure I – The crude precipitate was triturated with ethanol to give the title compound as a white powder (998 mg, 75% yield) after filtration.

TLC: *Rf* = 0.14 (1% 7 N NH_3 in methanol, 40% ethyl acetate and 59% diethyl ether).

^{1}H NMR (401 MHz, DMSO-*d*$_6$) *δ* 8.74 (br.s, 1H), 8.06 (s, 1H), 7.32 – 7.22 (m, 5H), 4.64 – 4.48 (m, 2H). Purine N*H* not observed.

2-Fluoro-*N*-(2-methoxyethyl)-9*H*-purin-6-amine (2b) [39]

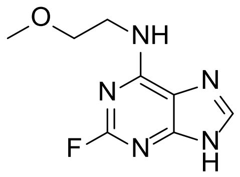

General reaction procedure I – The crude precipitate was recrystallised from isopropanol to give the title compound as a white powder (812 mg, 66% yield) after filtration.

TLC: *Rf* = 0.31 (2% 7 N NH_3 in methanol, 6% methanol and 92% dichloromethane).

^{1}H NMR (401 MHz, DMSO-*d*$_6$) *δ* 12.94 (s, 1H), 8.09 (s, 2H), 3.59 – 3.50 (m, 4H), 3.27 (s, 3H).

^{13}C NMR (101 MHz, DMSO-*d*$_6$) *δ* 159.8 (C), 157.8 (C), 70.2 (CH_2), 58.0 (CH_3). Missing two quaternary carbons and one aromatic CH.

HPLC: t_R = 2.94 min, > 95% purity (214 and 254 nm).

HRMS: (ESI+) calc. m/z for $[C_8H_{10}N_5O + H]^+$ 212.0942, found: 212.0946.

6-Chloro-*N*-(3-(trifluoromethyl)benzyl)-9*H*-purin-2-amine (5a) [28]

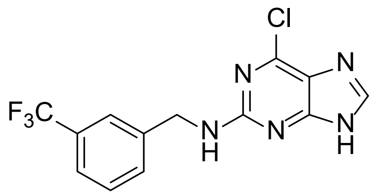

General procedure III – The concentrated residue was purified from ethanol recrystallisation and by column chromatography (20-70% ethyl acetate in dichloromethane) to give the title compound as a white powder (320 mg, 55% yield).

TLC: *Rf* = 0.33 (70% ethyl acetate in dichloromethane).

^{1}H NMR (401 MHz, DMSO-*d*$_6$) *δ* 12.98 (br.s, 1H), 8.11 (s, 1H), 8.00 (br.s, 1H), 7.68 (app.s, 1H), 7.65 – 7.61 (m, 1H), 7.59 – 7.52 (m, 2H), 4.59 (d, *J* = 6.2 Hz, 2H).

^{13}C NMR (101 MHz, DMSO-d_6) δ 158.7 (C), 141.6 (C), 131.1 (CH), 129.3 (CH), 129.1 (C), 128.8 (C), 125.6 (C), 123.5 (d, J = 3.9 Hz, CH), 123.3 (d, J = 3.7 Hz, CH), 122.9 (C), 44.1 (CH_2). One quaternary carbon and one aromatic CH was not observed.

HPLC: t_R = 5.01 min, > 97% purity (214 and 254 nm).

HRMS: (ESI+) calc. m/z for $[C_{13}H_9{}^{35}ClF_3N_5 + H]^+$ 328.0571, found: 328.058.

N-(3-Bromobenzyl)-6-chloro-9*H*-purin-2-amine (5b)

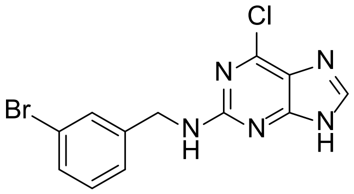

General procedure III – The concentrated residue was purified from ethanol recrystallisation and by column chromatography (2% 7 N NH_3 in methanol, 2% methanol and 96% dichloromethane) to give the title compound as a white powder (503 mg, 50% yield).

TLC: *Rf* = 0.30 (2% 7 N NH_3 in methanol, 2% methanol, 36% ethyl acetate and 60% dichloromethane).

^{1}H NMR (401 MHz, DMSO-d_6) δ 12.98 (br.s, 1H), 8.11 (s, 1H), 7.94 (br.s, 1H), 7.51 (t, J = 1.8 Hz, 1H), 7.41 (ddd, J = 7.8, 2.1, 1.3 Hz, 1H), 7.33 (dt, J = 7.7, 1.4 Hz, 1H), 7.26 (t, J = 7.7 Hz, 1H), 4.50 (d, J = 6.3 Hz, 2H).

^{13}C NMR (101 MHz, DMSO-d_6) δ 158.7 (C), 143.1 (C), 130.4 (CH), 129.6 (CH), 129.4 (CH), 126.0 (CH), 121.6 (C), 43.9 (CH_2). Three quaternary carbons and one aromatic CH was not observed.

HPLC: t_R = 4.95 min, > 96% purity (214 and 254 nm).

HRMS: (ESI+) calc. m/z for $[C_{12}H_9{}^{79}Br^{35}ClN_5 + H]^+$ 337.9803, found: 337.9806.

3-(((6-Chloro-9*H*-purin-2-yl)amino)methyl)phenol (5c)

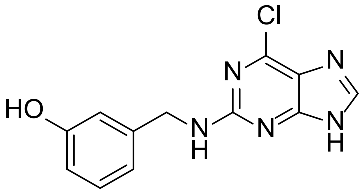

General procedure III – The concentrated residue was purified by column chromatography (1% 7 N NH_3 in methanol, 5% methanol and 94% dichloromethane) to give the title compound as a beige powder (105 mg, 64% yield).

TLC: *Rf* = 0.20 (1% 7 N NH_3 in methanol, 5% methanol and 94% dichloromethane).

^{1}H NMR (401 MHz, DMSO-*d*$_6$) δ 12.85 (br.s, 1H), 9.26 (br.s, 1H), 8.09 (s, 1H), 7.87 (br.s, 1H), 7.07 (t, *J* = 7.7 Hz, 1H), 6.75 – 6.70 (m, 2H), 6.58 (dd, *J* = 8.0, 2.4 Hz, 1H), 4.42 (d, *J* = 6.3 Hz, 2H).

^{13}C NMR (101 MHz, DMSO-*d*$_6$) δ 158.9 (C), 157.3 (C), 141.8 (CH), 141.6 (C), 129.1 (CH), 117.4 (CH), 113.6 (CH), 113.4 (CH), 44.3 (CH_2). Three quaternary carbons not observed.

HPLC: t_R = 3.47 min, > 95% purity (214 and 254 nm).

HRMS: (ESI+) calc. m/z for $[C_{12}H_{10}{}^{35}ClN_5O + H]^+$ 276.0647, found 276.0655.

***N*^{6}-Benzyl-*N*^{2}-(3-(trifluoromethyl)benzyl)-9*H*-purine-2,6-diamine (9) [1,3]**

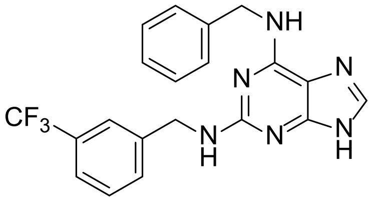

Method 1: General reaction procedure II from 2a – To the concentrated residue, was added cold diethyl ether and the title compound was collected as a white powder (12 mg, 30% yield) after filtration.

Method 2: **General reaction procedure IV from 5a –** To the concentrated residue was added cold dichloromethane. The precipitate was collected and was then washed with 0.5 M HCl followed by 30% ammonia solution. The title compound was collected as a white powder (60 mg, 70% yield).

TLC: *Rf* = 0.27 (1% 7 N NH_3 in methanol, 40% ethyl acetate and 59% diethyl ether).

^{1}H NMR (401 MHz, CD_3OD) δ 12.18 (br.s, 1H), 7.75 (br.s, 1H), 7.66 – 7.63 (m, 2H), 7.58 – 7.53 (m, 2H), 7.49 – 7.46 (m, 1H), 7.28 – 7.16 (m, 5H), 6.97 – 6.94 (m, 1H), 4.59 (app.br.s, 2H), 4.51 (d, *J* = 6.3 Hz, 2H).

^{13}C NMR (101 MHz, CD_3OD) δ 159.1 (C), 143.2 (C), 140.6 (C), 131.1 (CH), 129.0 (CH), 128.9 (C), 128.6 (C), 128.1 (CH), 127.4 (CH), 126.5 (CH), 125.8 (C), 123.4 (CH, q, *J* = 3.8 Hz), 123.1 (C), 123.0 (CH, q, *J* = 3.5 Hz), 44.1 (CH_2). One quaternary carbon and one aromatic CH not observed.

HPLC: t_R = 5.37 min, > 95% purity (214 and 254 nm).

HRMS: (ESI+) calc. m/z for $[C_{20}H_{17}F_3N_6 + H]^+$ 399.1540, found 399.1551.

***N*^{6}-Benzyl-*N*^{2}-(3-(difluoromethoxy)benzyl)-9*H*-purine-2,6-diamine (10)**

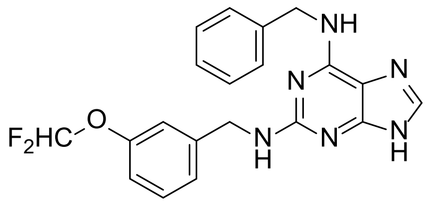

General reaction procedure II from 2a – To the concentrated residue, was added cold diethyl ether. The precipitate was filtered and washed with cold diethyl ether. It was then purified by column chromatography (20% ethyl acetate in diethyl ether) to give the title compound as a light-yellow powder (51 mg, 63% yield).

TLC: *Rf* = 0.24 (1% 7 N NH_3 in methanol, 40% ethyl acetate and 59% diethyl ether).

^{1}H NMR (401 MHz, $CDCl_3$ + a drop of CD_3OD) δ 7.49 (s, 1H), 7.30 – 7.19 (m, 6H), 7.15 – 7.12 (m, 1H), 7.07 (t, J = 2.0 Hz, 1H), 6.95 (dd, J = 8.1, 2.6 Hz, 1H), 6.43 (t, J = 74.1 Hz, 1H), 4.68 (br.s, 2H), 4.56 – 4.55 (m, 2H).

^{13}C NMR (101 MHz, $CDCl_3$ + a drop of CD_3OD) δ 152.2 (C), 151.4 (C), 140.1 (C), 130.1 (CH), 128.7 (CH), 127.7 (CH), 127.2 (CH), 124.1 (CH), 118.3 (CH), 118.2 (C), 44.9 (CH_2). Three quaternary carbons, two aromatic CH and O-CHF_2 not observed.

HPLC: t_R = 5.15 min, > 95% purity (214 and 254 nm).

HRMS: (ESI+) calc. m/z for $[C_{20}H_{18}F_2N_6O + H]^+$ 397.1583, found 397.1598.

***N*^{6}-Benzyl-*N*^{2}-(3-methylbenzyl)-9*H*-purine-2,6-diamine (11)**

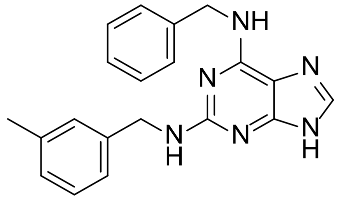

General reaction procedure II from 2a – To the concentrated residue, was added cold diethyl ether and the title compound was collected as a white powder (5.2 mg, 6.1% yield) after filtration.

TLC: *Rf* = 0.21 (1% 7 N NH_3 in methanol, 40% ethyl acetate and 59% diethyl ether).

^{1}H NMR (401 MHz, $CDCl_3$ + a drop of CD_3OD) δ 7.35 – 7.26 (m, 5H), 7.24 – 7.15 (m, 2H), 7.13 – 7.10 (m, 2H), 7.05 (d, J = 7.5 Hz, 1H), 4.72 (s, 2H), 4.59 – 4.55 (m, 2H), 2.30 (s, 3H).

^{13}C NMR (101 MHz, $CDCl_3$ + a drop of CD_3OD) δ 139.5 (C), 138.8 (C), 138.1 (C), 128.5 (CH), 128.4 (CH), 128.1 (CH), 127.8 (CH), 127.6 (CH), 127.2 (CH), 124.4 (CH), 45.7 (CH_2), 21.3 (CH_3). Four quaternary carbons and one aromatic CH not observed.

HPLC: t_R = 5.20 min, > 95% purity (214 and 254 nm).

HRMS: (ESI+) calc. m/z for $[C_{19}H_{19}N_7 + H]^+$ 345.1822, found 345.1831.

N^6**-Benzyl-*N***^{2}**-(3-(trifluoromethoxy)benzyl)-9*H*-purine-2,6-diamine (12)**

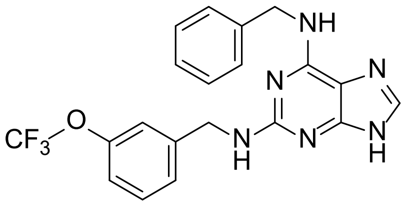

General reaction procedure II from 2a – To the concentrated residue, was added dichloromethane and petroleum ether. The precipitate was collected and was then washed with 0.5 M HCl followed by 30% ammonia solution. The title compound was collected as a white powder (19 mg, 19% yield).

TLC: *Rf* = 0.17 (1% 7 N NH_3 in methanol and 99% dichloromethane).

^{1}H NMR (401 MHz, DMSO-*d*$_6$) δ 12.18 (br.s, 1H), 7.78 (br.s, 1H), 7.65 (s, 1H), 7.38 (t, *J* = 7.9 Hz, 1H), 7.30 – 7.28 (m, 3H), 7.26 – 7.24 (m, 3H), 7.19 – 7.15 (m, 2H), 6.94 – 6.91 (m, 1H), 4.59 (br.s, 2H), 4.47 (d, *J* = 6.4 Hz, 2H).

^{13}C NMR (101 MHz, DMSO-*d*$_6$) δ 159.1 (C), 148.4 (C), 144.7 (C), 129.9 (CH), 128.0 (CH), 127.4 (CH), 126.4 (CH), 126.0 (CH), 121.4 (C), 119.2 (CH), 118.8 (C), 118.6 (CH), 44.0 (CH_2). Three quaternary carbons and one aromatic CH not observed.

HPLC: t_R = 5.45 min, > 93% purity (214 and 254 nm).

HRMS: (ESI+) calc. m/z for $[C_{20}H_{17}N_6O + H]^+$ 415.1489, found 415.1496.

N^6**-Benzyl-*N***^{2}**-(3-methoxybenzyl)-9*H*-purine-2,6-diamine (13)**

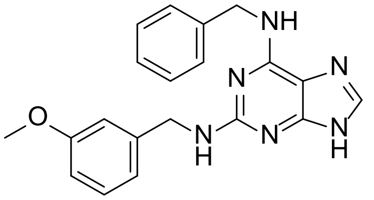

General reaction procedure II from 2a – To the concentrated residue, was added cold diethyl ether. The precipitate was filtered and washed with cold diethyl ether. It was then purified by column

chromatography (2% 7 N NH_3 in methanol, 2% methanol and 96% diethyl ether) to give the title compound as a white powder (8.3 mg, 11% yield).

TLC: *Rf* = 0.17 (1% 7 N NH_3 in methanol, 40% ethyl acetate and 59% diethyl ether).

^{1}H NMR (401 MHz, DMSO-*d*$_6$) δ 12.16 (br.s, 1H), 7.73 (br.s, 1H), 7.64 (s, 1H), 7.32 (d, *J* = 7.5 Hz, 2H), 7.25 (t, *J* = 7.4 Hz, 2H), 7.20 – 7.14 (m, 2H), 6.88 – 6.85 (m, 2H), 6.79 – 6.72 (m, 2H), 4.62 (br.s, 2H), 4.42 (d, *J* = 6.3 Hz, 2H), 3.69 (s, 3H).

^{13}C NMR (101 MHz, DMSO-*d*$_6$) δ 159.3 (C), 159.2 (C), 143.2 (C), 140.7 (C), 135.2 (CH), 129.0 (CH), 128.0 (CH), 127.4 (CH), 126.4 (CH), 119.2 (CH), 112.7 (CH), 111.4 (CH), 54.9 (CH_3), 44.4 (CH_2). Three quaternary carbons and one aliphatic CH_2 not observed.

HPLC: t_R = 4.95 min, > 95% purity (214 and 254 nm).

HRMS: (ESI+) calc. m/z for $[C_{19}H_{19}N_7 + H]^+$ 361.1771, found 361.1772.

N^6-Benzyl-*N*^{2}-(3-bromobenzyl)-9*H*-purine-2,6-diamine (14)

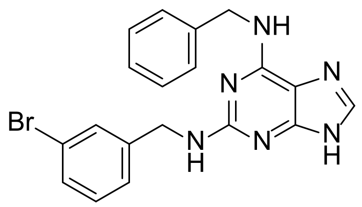

General procedure IV from 5b – The concentrated residue was added cold dichloromethane. The precipitate was collected and was then washed with 1 M HCl followed by 30% ammonia solution. The title compound was collected as a beige powder (107 mg, 89% yield).

TLC: *Rf* = 0.30 (1% 7 N NH_3 in methanol, 2% methanol and 97% dichloromethane).

^{1}H NMR (401 MHz, DMSO-*d*$_6$) δ 12.23 (br.s, 1H), 7.81 (br.s, 1H), 7.68 (s, 1H), 7.47 – 7.46 (m, 1H), 7.37 (dt, *J* = 7.7, 1.6 Hz, 1H), 7.31 – 7.17 (m, 7H), 7.03 – 6.94 (m, 1H), 4.60 (br.s, 2H), 4.43 (d, *J* = 6.3 Hz, 2H).

^{13}C NMR (101 MHz, DMSO-*d*$_6$) δ 130.2 (CH), 129.6 (CH), 129.0 (CH), 128.1 (CH), 127.4 (CH), 126.5 (CH), 126.0 (CH), 121.5 (C), 43.9 (CH_2). Six quaternary carbons and one aromatic CH not observed.

HPLC: t_R = 5.31 min, > 96% purity (214 and 254 nm).

HRMS: (ESI+) calc. m/z for $[C_{19}H_{17}{}^{79}BrN_6 + H]^+$ 409.0771, found: 409.0781.

N^2-(3-(Aminomethyl)benzyl)-*N*^{6}-benzyl-9*H*-purine-2,6-diamine (15) [34]

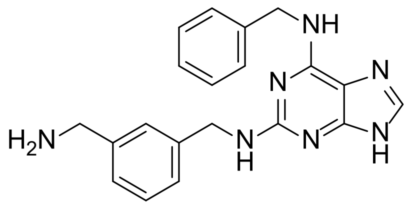

General reaction procedure II from 2a – The concentrated residue was added 1 M HCl. It was washed with dichloromethane (15 mL). The aqueous layer was added 1 M NaOH till pH ~ 10 and extracted with dichloromethane (3 x 15 mL). The organic extracts were combined, washed with brine, dried over $MgSO_4$ and concentrated *in vacuo*. The crude material was recrystallised from chloroform to give the title compound as a yellow powder (13 mg, 14%).

TLC: *Rf* = 0.10 (2% 7 N NH_3 in methanol, 8% methanol and 90% dichloromethane).

^{1}H NMR (401 MHz, DMSO-*d*$_6$) δ 7.69 – 7.64 (m, 2H), 7.34 – 7.11 (m, 10H), 6.72 (t, *J* = 6.5 Hz, 1H), 4.62 (br.s, 2H), 4.43 (d, *J* = 6.3 Hz, 1H), 3.66 (app.s, 2H).

^{13}C NMR (101 MHz, DMSO-*d*$_6$) δ 159.3 (C), 143.5 (C), 141.2 (C), 128.1 (CH), 127.8 (CH), 127.4 (CH), 126.4 (CH), 125.8 (CH), 125.0 (CH), 45.6 (CH_2), 44.5 (CH_2). Four quaternary carbons, two aromatic CH and one aliphatic CH_2 not observed.

HPLC: t_R = 3.81 min, > 95% purity (214 and 254 nm).

HRMS: (ESI+) calc. m/z for $[C_{20}H_{21}N_7 + H]^+$ 360.1931, found 360.1936.

Methyl 3-(((6-(benzylamino)-9*H*-purin-2-yl)amino)methyl)benzoate (16)

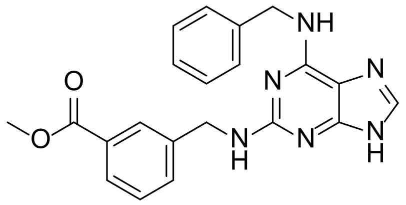

General reaction procedure II from 2a – The concentrated residue was added 0.5 M HCl. The precipitate was washed with 30% NH_4OH. The precipitate was then triturated with methanol to give the title product as a white powder (154 mg, 64% yield) after a second filtration.

TLC: *Rf* = 0.17 (1% 7 N NH_3 in methanol and 99% dichloromethane).

^{1}H NMR (401 MHz, $CDCl_3$ + a drop of CD_3OD) δ 7.97 – 7.96 (m, 1H), 7.85 (dt, *J* = 7.7, 1.4 Hz, 1H), 7.49 – 7.45 (m, 2H), 7.30 (t, *J* = 7.7 Hz, 1H), 7.25 – 7.18 (m, 5H), 4.66 (s, 2H), 4.57 (s, 2H), 3.83 (s, 3H).

^{13}C NMR (101 MHz, $CDCl_3$ + a drop of CD_3OD) δ 167.4 (C), 140.5 (C), 138.9 (C), 132.1 (CH), 130.2 (C), 128.6 (CH), 128.6 (CH), 128.3 (CH), 127.7 (CH), 127.2 (CH), 52.2 (CH_3), 45.5 (CH_2). Four quaternary carbons and two aromatic CH not observed.

HPLC: t_R = 4.96 min, > 95% purity (214 and 254 nm).

HRMS: (ESI+) calc. m/z for $[C_{21}H_{20}N_6O_2 + H]^+$ 389.1721, found 389.1731.

3-(((6-(Benzylamino)-9*H*-purin-2-yl)amino)methyl)benzoic acid (17)

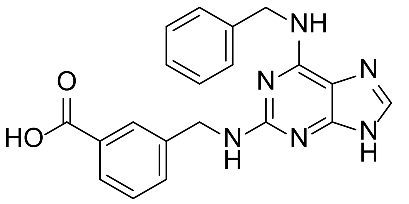

To a solution of methyl 3-(((6-(benzylamino)-9*H*-purin-2-yl)amino)methyl)benzoate (**16**, 101 mg, 257 µmol) in a mixture of tetrahydrofuran and water (1:1, 4 mL), was added lithium hydroxide monohydrate (27 mg, 644 µmol). The mixture warmed to 60 °C and stirred for 1 hour. After the consumption of starting material, it was concentrated *in vacuo*. It was then redissolved in water (10 mL) and washed with ethyl acetate (10 mL). The aqueous extract was acidified with 1 M HCl and the title compound was collected as a white powder precipitate (83 mg, 86% yield).

TLC: *Rf* = 0.25 (1% acetic acid, 5% methanol and 94% dichloromethane).

^{1}H NMR (401 MHz, DMSO-d_6) δ 12.83 (br.s, 1H), 12.17 (br.s, 1H), 7.91 (s, 1H), 7.77 – 7.74 (m, 2H), 7.65 (s, 1H), 7.51 (d, J = 7.7 Hz, 1H), 7.37 (t, J = 7.6 Hz, 1H), 7.31 – 7.16 (m, 5H), 6.94 (s, 1H), 4.60 – 4.48 (m, 4H).

^{13}C NMR (101 MHz, DMSO-d_6) δ 167.4 (C), 142.2 (C), 131.5 (CH), 130.5 (C), 128.2 (CH), 128.1 (CH), 127.8 (CH), 127.4 (CH), 127.2 (CH), 126.4 (CH), 44.1 (CH_2). Five quaternary carbons and one aromatic CH not observed.

HPLC: t_R = 4.41 min, > 98% purity (214 and 254 nm).

HRMS: (ESI+) calc. m/z for $[C_{20}H_{18}N_6O_2 + H]^+$ 375.1564, found 375.1575.

N^6-Benzyl-*N*^{2}-(2-(trifluoromethyl)benzyl)-9*H*-purine-2,6-diamine (18)

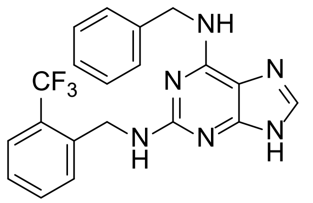

General reaction procedure II from 2a – To the concentrated residue, was added cold diethyl ether and the title compound was collected as a white powder (6.3 mg, 6.4% yield) after filtration.

TLC: *Rf* = 0.23 (1% 7 N NH_3 in methanol, 40% ethyl acetate and 59% diethyl ether).

^{1}H NMR (401 MHz, $CDCl_3$ + a drop of CD_3OD) δ 7.68 (s, 1H), 7.62 (d, J = 7.8 Hz, 1H), 7.56 (d, J = 7.8 Hz, 1H), 7.46 (t, J = 7.6 Hz, 1H), 7.34 (t, J = 7.6 Hz, 1H), 7.24 – 7.15 (m, 5H), 4.80 (s, 2H), 4.62 (app. d, J = 12.6 Hz, 2H).

^{13}C NMR (101 MHz, CD_3OD) δ 164.6 (C), 140.9 (C), 140.9 (C), 138.1 (C), 133.2 (CH), 129.4 (CH), 129.4 (CH), 128.5 (CH), 127.9 (CH), 127.7 (CH), 127.6 (C), 126.7 (CH), 126.6 (CH), 42.9 (CH_2). Three quaternary carbons not observed.

HPLC: t_R = 5.34 min, > 95% purity (214 and 254 nm).

HRMS: (ESI+) calc. m/z for $[C_{19}H_{19}N_7 + H]^+$ 399.1540, found 399.1551.

N[6]-Benzyl-*N*[2]-(4-(trifluoromethyl)benzyl)-9*H*-purine-2,6-diamine (19)

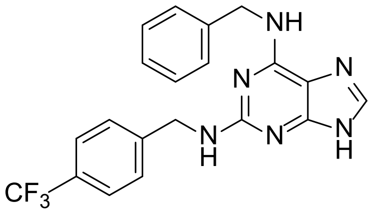

General reaction procedure II from 2a – To the concentrated residue, was added cold diethyl ether and the title compound was collected as a white powder (16 mg, 16% yield) after filtration.

TLC: *Rf* = 0.19 (1% 7 N NH_3 in methanol, 40% ethyl acetate and 59% diethyl ether).

^{1}H NMR (401 MHz, $CDCl_3$ + a drop of CD_3OD) δ 7.62 (s, 1H), 7.48 (d, J = 8.2 Hz, 2H), 7.42 (d, J = 8.1 Hz, 2H), 7.25 – 7.16 (m, 5H), 4.66 (s, 2H), 4.62 (s, 2H).

^{13}C NMR (101 MHz, $CDCl_3$ + CD_3OD) δ 154.9 (C), 145.1 (C), 139.4 (C), 129.4 (C), 129.1 (C), 128.8 (CH), 127.8 (CH), 127.7 (CH), 127.5 (CH), 126.1 (C), 125.6 (CH), 125.53 (CH), 123.4 (C), 121.1 (C), 45.5 (CH_2).

HPLC: t_R = 5.40 min, > 95% purity (214 and 254 nm).

HRMS: (ESI+) calc. m/z for $[C_{19}H_{19}N_7 + H]^+$ 399.1540, found 399.1557.

N[6]-Benzyl-*N*[2]-(2-(trifluoromethoxy)benzyl)-9*H*-purine-2,6-diamine (20)

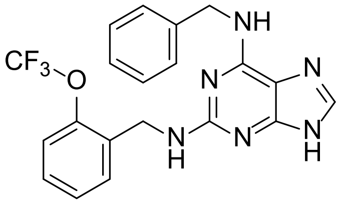

General reaction procedure II from 2a – The concentrated residue was added 0.5 M HCl. The precipitate was washed with 30% NH_4OH. The precipitate was then triturated with methanol to give the title product as a white powder (179 mg, 70% yield)

TLC: *Rf* = 0.16 (1% 7 N NH_3 in methanol and 99% dichloromethane).

^{1}H NMR (401 MHz, DMSO-*d*$_6$) δ 7.93 (br.s, 1H), 7.42 – 7.18 (m, 11H), 7.07 (s, 1H), 4.76 – 4.59 (m, 4H).

^{13}C NMR (101 MHz, DMSO-*d*$_6$) δ 146.1 (C), 128.7 (CH), 128.1 (CH), 128.0 (CH), 127.5 (CH), 127.3 (CH), 126.6 (CH), 121.6 (C), 120.7 (C), 120.4 (CH), 119.0 (C). Four quaternary carbons, one aromatic CH and aliphatic CH_2 not observed.

HPLC: t_R = 5.42 min, > 91% purity (214 and 254 nm).

HRMS: (ESI+) calc. m/z for $[C_{20}H_{17}N_6O + H]^+$ 415.1489, found 415.1499.

N[6]-Benzyl-*N*[2]-(4-(trifluoromethoxy)benzyl)-9*H*-purine-2,6-diamine (22)

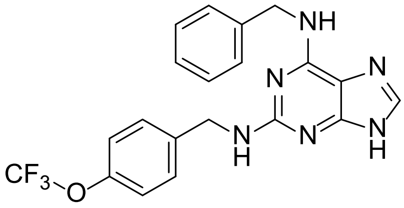

General reaction procedure II from 2a – To the concentrated residue, was added dichloromethane and petroleum ether. The precipitate was collected and was then washed with 0.5 M HCl followed by 30% ammonia solution. The title compound was collected as a white powder (28 mg, 27% yield).

TLC: *Rf* = 0.14 (1% 7 N NH_3 in methanol and 99% dichloromethane).

^{1}H NMR (401 MHz, DMSO-*d*$_6$) δ 12.17 (br.s, 1H), 7.77 (br.s, 1H), 7.65 (s, 1H), 7.37 (d, *J* = 8.3 Hz, 2H), 7.31 – 7.29 (m, 2H), 7.26 – 7.16 (m, 5H), 6.90 – 6.88 (m, 1H), 4.60 (s, 2H), 4.45 (d, *J* = 6.3 Hz, 2H).

^{13}C NMR (101 MHz, DMSO-d_6) δ 159.2 (C), 146.7 (C), 141.1 (C), 128.7 (CH), 128.0 (CH), 127.3 (CH), 126.4 (CH), 121.4 (C), 120.6 (CH), 43.8 (CH_2). Four quaternary carbons and one aromatic CH not observed.

HPLC: t_R = 5.50 min, > 91% purity (214 and 254 nm).

HRMS: (ESI+) calc. m/z for $[C_{20}H_{17}N_6O + H]^+$ 415.1489, found 415.1494.

N^6-Benzyl-N^2-(4-fluorobenzyl)-9H-purine-2,6-diamine (23) [34]

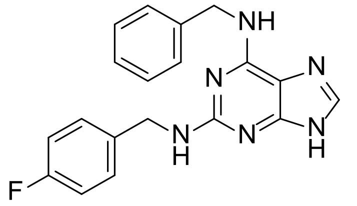

General reaction procedure II from 2a – To the concentrated residue, was added cold diethyl ether and the title compound was collected as a white powder (26 mg, 36% yield) after filtration.

TLC: *Rf* = 0.18 (1% 7 N NH_3 in methanol, 40% ethyl acetate and 59% diethyl ether).

^{1}H NMR (401 MHz, $CDCl_3$) δ 7.45 (s, 1H), 7.29 – 7.19 (m, 7H), 6.91 (t, J = 8.7 Hz, 2H), 4.69 – 4.64 (m, 2H), 4.56 – 4.51 (m, 2H).

^{13}C NMR (101 MHz, $CDCl_3$ + drop of CD_3OD) δ 163.1 (C), 160.7 (C), 154.6 (C), 138.9 (C), 135.6 (C), 135.3 (CH), 128.9 (d, J = 7.8 Hz, CH), 128.5 (CH), 127.5 (CH), 127.2 (CH), 115.2 (d, J = 21.6 Hz, CH), 45.0 (CH_2). Two quaternary carbons not observed.

HPLC: t_R = 5.02 min, > 95% purity (214 and 254 nm).

HRMS: (ESI+) calc. m/z for $[C_{19}H_{17}FN_6 + H]^+$ 349.1571, found 349.1586.

N^6-Benzyl-N^2-(2-methoxybenzyl)-9H-purine-2,6-diamine (24)

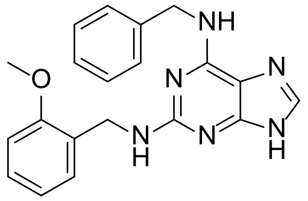

General reaction procedure II from 2a – The concentrated residue was purified by column chromatography (2% 7 N NH_3 in methanol, 3% methanol and 95% dichloromethane). It was then triturated with a mixture of dichloromethane and petroleum ether to give the title compound as a beige powder (8.8 mg, 9.9% yield) after filtration.

TLC: *Rf* = 0.17 (1% 7 N NH_3 in methanol, 40% ethyl acetate and 59% diethyl ether).

^{1}H NMR (401 MHz, $CDCl_3$) δ 7.35 – 7.18 (m, 8H), 6.89 – 6.85 (m, 2H), 4.77 (br.s, 2H), 4.65 (app. d, *J* = 5.7 Hz, 2H), 3.84 – 3.82 (m, 3H).

^{13}C NMR (101 MHz, $CDCl_3$) δ 157.5 (C), 128.8 (CH), 128.7 (CH), 128.5 (CH), 128.0 (CH), 127.4 (CH), 120.7 (CH), 110.3 (CH), 55.4 (CH_3), 41.4 (CH_2). Six quaternary carbons and one CH not observed.

HPLC: t_R = 5.11 min, > 95% purity (214 and 254 nm).

HRMS: (ESI+) calc. m/z for $[C_{19}H_{19}N_7 + H]^+$ 361.1771, found 361.1778.

N^6-Benzyl-*N*^{2}-(pyrimidin-2-ylmethyl)-9*H*-purine-2,6-diamine (25)

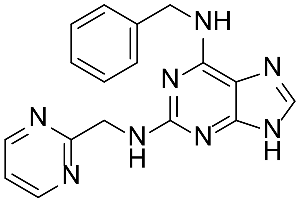

General reaction procedure II from 2a – The concentrated residue was purified by column chromatography (2% 7 N NH_3 in methanol, 5% methanol and 93% dichloromethane). It was then triturated with dichloromethane to give the title compound as a yellow powder (10 mg, 7.3% yield) after filtration.

TLC: *Rf* = 0.21 (2% 7 N NH_3 in methanol, 5% methanol and 93% dichloromethane).

^{1}H NMR (401 MHz, DMSO-*d*$_6$) δ 12.18 (s, 1H), 8.71 (d, *J* = 4.9 Hz, 2H), 7.71 (s, 1H), 7.64 (s, 1H), 7.33 – 7.16 (m, 6H), 6.57 (s, 1H), 4.63 (d, *J* = 6.0 Hz, 4H).

^{13}C NMR (101 MHz, DMSO-*d*$_6$) δ 159.1 (C), 157.1 (CH), 135.3 (CH), 128.0 (CH), 127.4 (CH), 126.4 (CH), 119.4 (CH), 47.3 (CH_2). Five quaternary carbons not observed.

HPLC: t_R = 3.88 min, > 95% purity (214 and 254 nm).

HRMS: (ESI+) calc. m/z for $[C_{17}H_{16}N_8 + H]^+$ 333.1571, found: 333.1578.

N^6-Benzyl-*N*^{2}-((1-methyl-1*H*-imidazol-2-yl)methyl)-9*H*-purine-2,6-diamine (26)

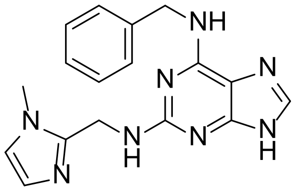

General reaction procedure II from 2a – To the concentrated residue, was added 0.5 M HCl and the insoluble compounds were filtered. The filtrate was made basic to pH ~ 10 and extracted with dichloromethane (3 x 10 mL). The organic extracts were combined, washed with brine, dried over $MgSO_4$ and concentrated *in vacuo*. The crude material was triturated with a mixture of dichloromethane and petroleum ether to give the title product as a brown powder (2.3 mg, 2% yield).

TLC: *Rf* = 0.30 (1% 7 N NH_3 in methanol, 5% methanol and 94% dichloromethane)

^{1}H NMR (401 MHz, DMSO-*d*$_6$) δ 12.25 (s, 1H), 7.80 (br.s, 1H), 7.68 (s, 1H), 7.36 (d, *J* = 7.1 Hz, 2H), 7.27 (t, *J* = 7.5 Hz, 2H), 7.21 – 7.17 (m, 1H), 7.02 (d, *J* = 1.2 Hz, 1H), 6.75 (d, *J* = 1.2 Hz, 1H), 6.44 (br.t, *J* = 5.0 Hz, 1H), 4.64 (s, 2H), 4.2a (d, *J* = 5.6 Hz, 2H), 3.57 (s, 3H).

^{13}C NMR (101 MHz, DMSO-*d*$_6$) δ 158.8 (C), 145.7 (C), 135.4 (CH), 128.1 (CH), 127.4 (CH), 126.4 (CH), 126.1 (CH), 121.5 (CH), 37.7 (CH_2), 32.3 (CH_3). Four quaternary carbons not observed.

HPLC: t_R = 3.20 min, > 95% purity (214 and 254 nm).

HRMS: (ESI+) calc. m/z for $[C_{17}H_{18}N_8 + H]^+$ 335.1727, found 335.1736.

N^6-Benzyl-*N*^{2}-((tetrahydro-2H-pyran-4-yl)methyl)-9*H*-purine-2,6-diamine (27)

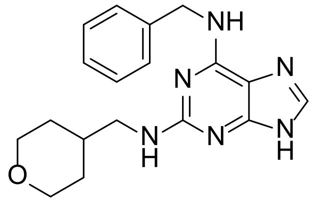

General reaction procedure II from 2a – The concentrated residue was purified by column chromatography (2% 7 N NH_3 in methanol, 5% methanol and 93% dichloromethane) to give the title compound as a beige powder (13 mg, 18% yield).

TLC: *Rf* = 0.46 (2% 7 N NH_3 in methanol, 5% methanol and 93% dichloromethane).

^{1}H NMR (401 MHz, DMSO-*d*$_6$) δ 12.12 (s, 1H), 7.68 – 7.63 (m, 2H), 7.35 – 7.32 (m, 2H), 7.29 – 7.26 (m, 2H), 7.21 – 7.17 (m, 1H), 6.27 (t, J = 5.9 Hz, 1H), 4.64 (br.s, 2H), 3.79 (d, *J* = 10.3 Hz, 2H), 3.17 (t, *J* = 11.6 Hz, 2H), 3.08 (t, *J* = 6.5 Hz, 2H), 1.74 (br.s, 1H), 1.53 (d, *J* = 13.2 Hz, 2H), 1.16 – 1.07 (m, 2H).

^{13}C NMR (101 MHz, DMSO-*d*$_6$) δ 159.6 (C), 135.1 (CH), 128.1 (CH), 127.2 (CH), 126.4 (CH), 66.9 (CH_2), 47.1 (CH_2), 30.8 (CH), 30.7 (CH_2). Four quaternary carbons and one CH_2 not observed.

HPLC: t_R = 4.37 min, > 95% purity (214 and 254 nm).

HRMS: (ESI+) calc. m/z for $[C_{18}H_{22}N_6O + H]^+$ 339.1928, found: 339.1935.

2-((6-(Benzylamino)-9*H*-purin-2-yl)amino)ethan-1-ol (28) [43]

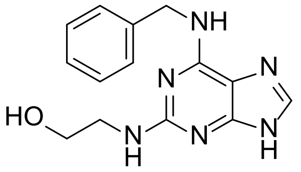

General reaction procedure II from 2a – The concentrated residue was purified by column chromatography (2% 7 N NH_3 in methanol, 10% methanol and 88% ethyl acetate) to give the title compound as a white powder (36 mg, 61% yield).

TLC: *Rf* = 0.25 (2% 7 N NH_3 in methanol, 10% methanol and 88% ethyl acetate).

^{1}H NMR (401 MHz, DMSO-*d*$_6$) δ 12.17 (s, 1H), 7.70 (br.s, 1H), 7.64 (s, 1H), 7.36 – 7.34 (m, 2H), 7.30 – 7.26 (m, 2H), 7.21 – 7.17 (m, 1H), 6.04 (t, *J* = 5.6 Hz, 1H), 4.65 – 4.63 (m, 3H), 3.49 (q, *J* = 5.7 Hz, 2H), 3.28 (q, *J* = 6.1 Hz, 2H).

^{13}C NMR (101 MHz, DMSO-*d*$_6$) δ 159.4 (C), 135.2 (CH), 128.1 (CH), 127.3 (CH), 126.4 (CH), 60.4 (CH_2), 43.9 (CH_2). Four quaternary carbons and one CH_2 not observed.

HPLC: t_R = 3.685 min, > 97% purity (214 and 254 nm).

HRMS: (ESI+) calc. m/z for $[C_{14}H_{16}N_6O + H]^+$ 285.1458, found 285.1466.

N^6-Benzyl-*N*^{2}-(2-methoxyethyl)-9*H*-purine-2,6-diamine (29)

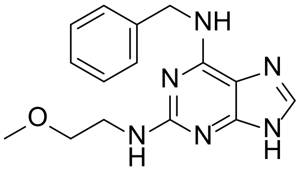

General reaction procedure II from 2a – To the concentrated residue, was added cold dichloromethane and the title compound was collected as a white powder (25 mg, 41% yield) after filtration.

TLC: *Rf* = 0.40 (1% 7 N NH_3 in methanol, 5% methanol and 94% dichloromethane).

^{1}H NMR (401 MHz, DMSO-*d*$_6$) δ 12.17 (s, 1H), 7.72 (br.s, 1H), 7.65 (s, 1H), 7.35 – 7.33 (m, 2H), 7.30 – 7.26 (m, 2H), 7.21 – 7.17 (m, 1H), 6.09 (t, *J* = 5.3 Hz, 1H), 4.63 (br.s, 2H), 3.39 – 3.35 (m, 4H), 3.21 (s, 3H).

^{13}C NMR (101 MHz, DMSO-*d*$_6$) δ 159.2 (C), 140.8 (C), 135.2 (CH), 128.1 (CH), 127.3 (CH), 126.4 (CH), 70.9 (CH_2), 57.9 (CH_3), 40.6 (CH_2). Three quaternary carbons and one CH2 not observed.

HPLC: t_R = 4.16 min, > 95% purity (214 and 254 nm).

HRMS: (ESI+) calc. m/z for $[C_{15}H_{18}N_6O + H]^+$ 299.1615, found: 299.1621.

***N*^{6}-(2-Methoxyethyl)-*N*^{2}-(3-(trifluoromethyl)benzyl)-9*H*-purine-2,6-diamine (30) [39]**

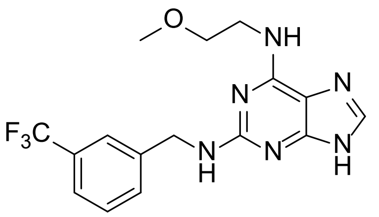

General procedure IV from 5a – The concentrated residue was added cold dichloromethane. The precipitate was collected and was then washed with 0.5 M HCl followed by 30% ammonia solution. The title compound was collected as a yellow powder (30 mg, 53% yield).

TLC: *Rf* = 0.40 (1% 7 N NH_3 in methanol, 5% methanol and 94% dichloromethane).

^{1}H NMR (401 MHz, DMSO-*d*$_6$) δ 12.21 (br.s, 1H), 7.67 – 7.65 (m, 2H), 7.63 – 7.60 (m, 1H), 7.56 – 7.49 (m, 2H), 7.14 – 7.02 (m, 2H), 4.53 (d, *J* = 6.3 Hz, 2H), 3.53 – 3.51 (m, 2H), 3.42 – 3.41 (m, 2H), 3.21 (s, 3H).

^{13}C NMR (101 MHz, DMSO-*d*$_6$) δ 143.2 (C), 131.2 (CH), 129.1 (CH), 129.0 (C), 128.7 (C), 125.8 (C), 123.4 (d, *J* = 4.0 Hz, CH), 123.1 (C), 123.0 (d, *J* = 3.9 Hz, CH), 70.6 (CH_2), 57.9 (CH_2), 57.9 (CH_3), 44.2 (CH_2). Two quaternary carbons and one aromatic CH not observed.

HPLC: t_R = 4.80 min, > 98% purity (214 and 254 nm).

HRMS: (ESI+) calc. m/z for $[C_{16}H_{17}F_3N_6O + H]^+$ 367.1489, found 367.1497.

***N*^{2}-(3-Bromobenzyl)-*N*^{6}-(2-methoxyethyl)-9*H*-purine-2,6-diamine (31)**

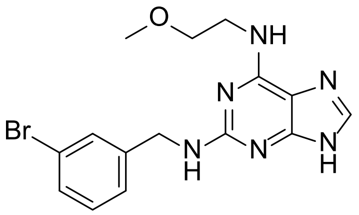

General procedure IV from 5b – The concentrated residue was added cold dichloromethane and the title compound was collected as a white powder (101 mg, 90% yield).

TLC: *Rf* = 0.18 (1% 7 N NH_3 in methanol, 2% methanol and 97% dichloromethane).

^{1}H NMR (401 MHz, DMSO-*d*$_6$) δ 12.16 (br.s, 1H), 7.64 (s, 1H), 7.49 (t, J = 1.8 Hz, 1H), 7.37 (ddd, J = 7.8, 2.1, 1.2 Hz, 1H), 7.31 (dt, J = 7.7, 1.3 Hz, 1H), 7.24 (t, J = 7.7 Hz, 1H), 7.04 (br.s, 1H), 6.91 (br.s, 1H), 4.44 (d, J = 6.4 Hz, 2H), 3.55 (app.br.s, 2H), 3.44 – 3.42 (m, 2H), 3.23 (s, 3H).

^{13}C NMR (101 MHz, DMSO-*d*$_6$) δ 130.4 (CH), 129.8 (CH), 129.4 (CH), 126.1 (CH), 121.5 (C), 70.1 (CH_2), 57.9 (CH_3), 43.9 (CH_2). Five quaternary carbons, one aromatic CH and one aliphatic CH_2 (under DMSO-*d*$_6$ peak) not observed.

HPLC: t_R = 4.61 min, > 98% purity (214 and 254 nm).

HRMS: (ESI+) calc. m/z for $[C_{15}H_{17}{}^{79}BrN_6O + H]^+$ 377.0720, found: 377.0727.

N^2-(3-Iodobenzyl)-*N*^{6}-(2-methoxyethyl)-9*H*-purine-2,6-diamine (32)

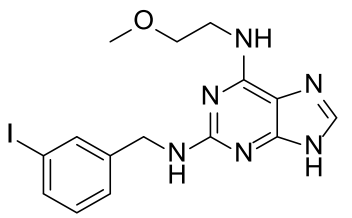

General reaction procedure II from 2b – The concentrated residue was purified by column chromatography (1% 7 N NH_3 in methanol, 2% methanol and 97% chloroform). It was then triturated with dichloromethane to give the title compound as an off-white powder (37 mg, 31% yield) after filtration.

TLC: *Rf* = 0.13 (1% 7 N NH_3 in methanol, 2% methanol and 97% chloroform).

^{1}H NMR (401 MHz, DMSO-*d*$_6$) δ 12.15 (s, 1H), 7.68 (t, J = 1.7 Hz, 1H), 7.63 (s, 1H), 7.54 (ddd, J = 7.8, 1.8, 1.1 Hz, 1H), 7.32 (dt, J = 7.9, 1.2 Hz, 1H), 7.10 – 7.03 (m, 2H), 6.89 (br.s, 1H), 4.40 (d, J = 6.4 Hz, 2H), 3.59 – 3.52 (m, 2H), 3.45 – 3.42 (m, 2H), 3.23 (s, 3H).

^{13}C NMR (101 MHz, DMSO-*d*$_6$) δ 159.1 (C), 144.5 (C), 135.5 (CH), 134.9 (CH), 130.3 (CH), 126.4 (CH), 119.6 (C), 94.6 (C), 70.6 (CH_2), 57.9 (CH_3), 43.9 (CH_2). Two quaternary carbons, one aromatic CH and one aliphatic CH_2 not observed.

HPLC: t_R = 4.90 min, > 96% purity (214 and 254 nm).

HRMS: (ESI+) calc. m/z for $[C_{15}H_{17}IN_6O + H]^+$ 425.0581, found 425.0592.

Methyl 3-(((6-((2-methoxyethyl)amino)-9*H*-purin-2-yl)amino)methyl)benzoate (33)

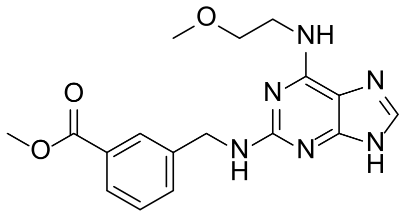

General reaction procedure II from 2b – The concentrated residue did not require further purification. The title compound was a yellow powder (734 mg, 55% yield).

TLC: *Rf* = 0.15 (2% 7 N NH_3 in methanol, 3% methanol and 95% dichloromethane).

^{1}H NMR (401 MHz, DMSO-*d*$_6$) δ 12.15 (br.s, 1H), 7.94 – 7.93 (m, 1H), 7.78 (dt, *J* = 7.7, 1.5 Hz, 1H), 7.63 – 7.57 (m, 2H), 7.43 (t, *J* = 7.7 Hz, 1H), 7.02 – 6.95 (m, 2H), 4.50 (d, *J* = 6.4 Hz, 2H), 3.82 (s, 3H), 3.54 (app.br.s, 2H), 3.43 (app.s, 2H), 3.22 (s, 3H).

^{13}C NMR (101 MHz, DMSO-*d*$_6$) δ 166.3 (C), 132.1 (CH), 129.6 (C), 128.7 (CH), 127.8 (CH), 127.4 (CH), 70.2 (CH_2), 57.9 (CH_3), 52.1 (CH_3), 44.2 (CH_2). Five quaternary carbons, one aromatic CH and one aliphatic CH_2 not observed.

HPLC: t_R = 4.12 min, > 98% purity (214 and 254 nm).

HRMS: (ESI+) calc. m/z for $[C_{17}H_{20}N_6O_3 + H]^+$ 357.1670, found 357.1680.

3-(((6-((2-Methoxyethyl)amino)-9*H*-purin-2-yl)amino)methyl)benzoic acid (34)

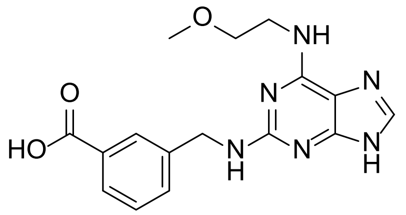

To a solution of methyl 3-(((6-((2-methoxyethyl)amino)-9*H*-purin-2-yl)amino)methyl)benzoate (**33**, 500 mg, 1.4 mmol) in a mixture of tetrahydrofuran and water (1:1, 4 mL), was added lithium hydroxide monohydrate (27 mg, 644 µmol). The mixture warmed to 60 °C and stirred for 1 hour. After the consumption of starting material, it was concentrated *in vacuo*. It was then redissolved in water (10 mL) and washed with ethyl acetate (10 mL). The aqueous extract was acidified with 1 M HCl and the title compound was collected as a white powder precipitate (388 mg, 81% yield).

TLC: *Rf* = 0.51 (1% acetic acid, 10% methanol and 89% dichloromethane).

^{1}H NMR (401 MHz, DMSO-*d*$_6$) δ 12.16 (br.s, 1H), 7.91 (br.t, J = 1.8 Hz, 1H), 7.76 (dt, J = 7.7, 1.5 Hz, 1H), 7.64 (s, 1H), 7.55 (dt, J = 7.7, 1.5 Hz, 1H), 7.40 (t, J = 7.6 Hz, 1H), 7.01 – 6.93 (m, 2H), 4.50 (d, J = 6.4 Hz, 2H), 3.55 – 3.41 (m, 4H), 3.22 (s, 3H).

^{13}C NMR (101 MHz, DMSO-*d*$_6$) δ 167.5 (C), 159.2 (C), 142.2 (C), 131.5 (CH), 130.6 (C), 128.2 (CH), 127.8 (CH), 127.2 (CH), 70.6 (CH_2), 57.8 (CH_3), 44.2 (CH_2).

HPLC: t_R = 3.63 min, > 99% purity (214 and 254 nm).

HRMS: (ESI+) calc. m/z for $[C_{16}H_{18}N_6O_3 + H]^+$ 343.1513, found 343.1522.

N^6-(2-Methoxyethyl)-*N*^{2}-(pyridin-3-ylmethyl)-9*H*-purine-2,6-diamine (35)

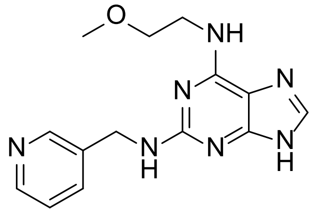

General reaction procedure II from 2b – The concentrated residue was purified by column chromatography (1% 7 N NH_3 in methanol, 5% methanol and 94% dichloromethane). It was then triturated with dichloromethane to give the title compound as an off-white powder (54 mg, 63% yield) after filtration.

TLC: *Rf* = 0.50 (1% 7 N NH_3 in methanol, 5% methanol and 94% dichloromethane).

^{1}H NMR (401 MHz, DMSO-*d*$_6$) δ 12.16 (br.s, 1H), 8.53 (d, J = 2.2 Hz, 1H), 8.39 (dd, J = 4.8, 1.7 Hz, 1H), 7.70 (dt, J = 7.9, 2.0 Hz, 1H), 7.64 (s, 1H), 7.29 (ddd, J = 7.8, 4.8, 0.9 Hz, 1H), 7.03 (br.s, 1H), 6.90 (br.s, 1H), 4.45 (d, J = 6.3 Hz, 2H), 3.55 (app.br.s, 2H), 3.45 – 3.42 (m, 2H), 3.23 (s, 3H).

^{13}C NMR (101 MHz, DMSO-*d*$_6$) δ 159.1 (C), 148.7 (CH), 147.5 (CH), 136.8 (C), 135.3 (CH), 134.8 (CH), 123.3 (CH), 70.6 (CH_2), 57.9 (CH_3), 42.2 (CH_2). Three quaternary carbons and one aliphatic CH_2 not observed.

HPLC: t_R = 2.82 min, > 97% purity (214 and 254 nm).

HRMS: (ESI+) calc. m/z for $[C_{14}H_{17}N_7O + H]^+$ 300.1567, found 300.1574.

3-(((6-((2-Methoxyethyl)amino)-9*H*-purin-2-yl)amino)methyl)phenol (36)

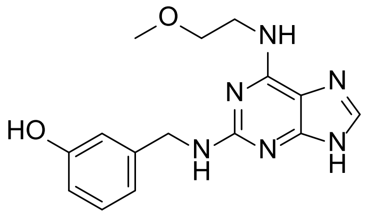

General procedure IV from 5c – The concentrated residue was added cold dichloromethane and the title compound was collected as a white powder (64 mg, 56% yield).

TLC: *Rf* = 0.30 (1% 7 N NH_3 in methanol, 7% methanol and 92% dichloromethane).

^{1}H NMR (401 MHz, DMSO-*d*$_6$) δ 12.16 (s, 1H), 9.19 (s, 1H), 7.64 (s, 1H), 7.04 (t, J = 8.0 Hz, 2H), 6.75 – 6.70 (m, 3H), 6.57 – 6.54 (m, 1H), 4.38 (d, J = 6.3 Hz, 2H), 3.56 – 3.44 (m, 4H), 3.23 (s, 3H).

^{13}C NMR (101 MHz, DMSO-*d*$_6$) δ 159.3 (C), 157.2 (C), 143.1 (C), 135.2 (CH), 128.9 (CH), 117.5 (CH), 113.7 (CH), 113.1 (CH), 70.6 (CH_2), 57.9 (CH_3), 44.3 (CH_2). Three quaternary carbons and one aliphatic CH_2 not observed.

HPLC: t_R = 3.59 min, > 95% purity (214 and 254 nm).

HRMS: (ESI+) calc. m/z for $[C_{15}H_{18}N_6O_2 + H]^+$ 315.1564, found 315.1575.

4-((6-((2-Methoxyethyl)amino)-9*H*-purin-2-yl)amino)phenol (37)

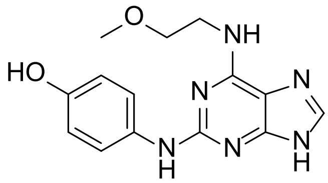

To a solution of 2-fluoro-*N*-(2-methoxyethyl)-9*H*-purin-6-amine (**2b**, 60 mg, 284 µmol) in *n*-butanol (1 mL), was added 4-aminophenol (46 mg, 426 µmol) and trifluoroacetic acid (42 mg, 28 µL, 369 µmol). The reaction was heated to reflux and stirred overnight (16 hours). It was then cooled to room temperature and concentrated *in vacuo*. The residue was purified by column chromatography (1% 7 N NH_3 in methanol, 5% methanol and 94% dichloromethane) to give the title compound as a brown powder (53 mg, 62% yield).

TLC: *Rf* = 0.28 (2% 7 N NH_3 in methanol, 2% methanol and 96% dichloromethane).

^{1}H NMR (401 MHz, DMSO-*d*$_6$) δ 12.31 (br.s, 1H), 8.87 (s, 1H), 8.45 (s, 1H), 7.74 (s, 1H), 7.53 (d, J = 8.6 Hz, 2H), 7.18 (br.s, 1H), 6.64 (d, J = 8.7 Hz, 2H), 3.63 (app.br.s, 2H), 3.53 (t, J = 5.9 Hz, 2H), 3.28 (s, 3H).

^{13}C NMR (101 MHz, DMSO-d_6) δ 156.7 (C), 151.3 (C), 135.9 (CH), 133.6 (C), 120.3 (CH), 114.8 (CH), 70.7 (CH_2), 58.0 (CH_3). Three quaternary carbons and one aliphatic CH_2 not observed.

HPLC: t_R = 3.21 min, > 95% purity (214 and 254 nm).

HRMS: (ESI+) calc. m/z for $[C_{14}H_{16}N_6O_2 + H]^+$ 301.1408, found 301.1419.

*N*²-(4-Methoxybenzyl)-*N*⁶-(2-methoxyethyl)-9*H*-purine-2,6-diamine (38)

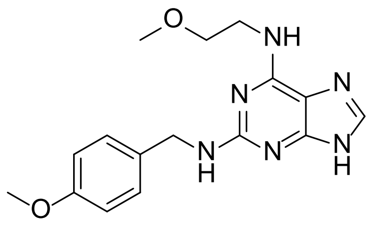

General reaction procedure II from 2b – To the concentrated residue, was added cold dichloromethane and the precipitate was collected. The precipitate was then washed with 1 M HCl followed by 30% ammonia solution. The title compound was collected as a yellow powder (25 mg, 27% yield).

TLC: *Rf* = 0.18 (2% 7 N NH_3 in methanol, 3% methanol and 95% dichloromethane).

^{1}H NMR (401 MHz, DMSO-d_6) δ 12.45 (br.s, 1H), 7.76 (s, 1H), 7.33 – 7.23 (m, 3H), 7.08 – 7.05 (m, 1H), 6.84 (d, J = 8.7 Hz, 2H), 4.40 (d, J = 6.2 Hz, 2H), 3.70 (s, 3H), 3.59 (app.br.s, 2H), 3.46 (t, J = 5.8 Hz, 2H), 3.24 (s, 3H).

^{13}C NMR (101 MHz, DMSO-d_6) δ 157.9 (C), 128.3 (CH), 113.5 (CH), 70.5 (CH_2), 57.9 (CH_3), 55.0 (CH_3), 43.9 (CH_2). Five quaternary carbons, one aromatic CH and one aliphatic CH2 not observed.

HPLC: t_R = 4.07 min, > 98% purity (214 and 254 nm).

HRMS: (ESI+) calc. m/z for $[C_{16}H_{20}N_6O_2 + H]^+$ 329.1721, found 329.1713.

*N*⁶-(2-Methoxyethyl)-*N*²-(4-methoxyphenethyl)-9*H*-purine-2,6-diamine (39)

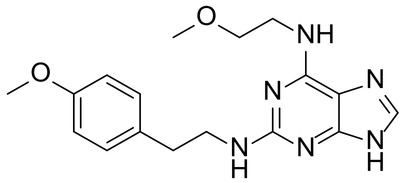

General reaction procedure II from 2b – The concentrated residue was purified by column chromatography (2% 7 N NH_3 in methanol, 2% methanol and 96% dichloromethane). It was then

triturated with dichloromethane to give the title compound as a white powder (43 mg, 44% yield) after filtration.

TLC: *Rf* = 0.30 (2% 7 N NH_3 in methanol, 2% methanol and 96% dichloromethane).

^{1}H NMR (401 MHz, DMSO-*d*$_6$) δ 12.14 (s, 1H), 7.63 (br.s, 1H), 7.15 (d, *J* = 8.6 Hz, 2H), 6.97 (br.s, 1H), 6.85 (d, *J* = 8.7 Hz, 2H), 6.19 (br.s, 1H), 3.72 (s, 3H), 3.60 (app.br.s, 2H), 3.50 (t, *J* = 5.8 Hz, 2H), 3.43 – 3.37 (m, 2H), 3.26 (s, 3H), 2.77 (dd, *J* = 8.6, 6.4 Hz, 2H).

^{13}C NMR (101 MHz, DMSO-*d*$_6$) δ 159.3 (C), 157.5 (C), 154.5 (C), 135.1 (CH), 132.0 (C), 129.5 (CH), 113.7 (CH), 70.7 (CH_2), 57.9 (CH_3), 55.0 (CH_3), 43.2 (CH_2), 34.6 (CH_2). Two quaternary carbons and one aliphatic CH_2 not observed.

HPLC: t_R = 4.31 min, > 98% purity (214 and 254 nm).

HRMS: (ESI+) calc. m/z for $[C_{17}H_{22}N_6O_2 + H]^+$ 343.1877, found 343.1886.

N^6-(2-Methoxyethyl)-*N*^{2}-(4-methoxyphenyl)-9*H*-purine-2,6-diamine (40)

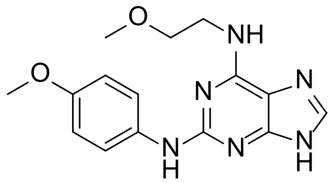

To a solution of 2-fluoro-*N*-(2-methoxyethyl)-9*H*-purin-6-amine (**2b**, 60 mg, 284 µmol) in *n*-butanol (1 mL), was added 4-methoxyaniline (245 mg, 229 µL, 2.0 mmol) and trifluoroacetic acid (42 mg, 28 µL, 369 µmol). The reaction was heated to reflux and stirred overnight (16 hours). It was then cooled to room temperature and the precipitate formed was collected, using cold *n*-butanol. The collected precipitate was triturated with methanol to give the title compound as a grey powder (21 mg, 24% yield) after filtration.

TLC: *Rf* = 0.31 (5% methanol in dichloromethane).

^{1}H NMR (401 MHz, DMSO-*d*$_6$) δ 8.95 (br.s, 1H), 8.02 (br.s, 1H), 7.70 – 7.60 (m, 3H), 6.85 (d, J = 9.0 Hz, 2H), 3.72 (s, 3H), 3.66 (br.s, 2H), 3.54 (t, J = 5.7 Hz, 2H), 3.29 (s, 3H). Purine NH not observed.

^{13}C NMR (101 MHz, DMSO-*d*$_6$) δ 154.1 (C), 153.3 (C), 120.9 (CH), 113.7 (CH), 70.4 (CH_2), 58.0 (CH_3), 55.2 (CH_3). Four quaternary carbons, one aromatic CH and one aliphatic CH_2 not observed.

HPLC: t_R = 3.88 min, > 96% purity (214 and 254 nm).

HRMS: (ESI+) calc. m/z for $[C_{15}H_{18}N_6O_2 + H]^+$ 315.1564, found 315.1572.

6-Morpholino-*N*-(3-(trifluoromethyl)benzyl)-9*H*-purin-2-amine (41)

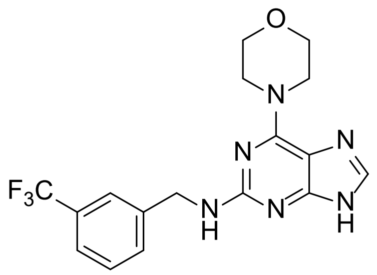

General procedure IV from 5a – The concentrated residue was purified by column chromatography (1% 7 N NH_3 in methanol, 2% methanol and 97% dichloromethane) to give the title compound as an off-white powder (52 mg, 90% yield).

TLC: *Rf* = 0.28 (1% 7 N NH_3 in methanol, 2% methanol and 97% dichloromethane).

^{1}H NMR (401 MHz, DMSO-*d*$_6$) δ 12.29 (s, 1H), 7.69 (s, 1H), 7.66 (br.s, 1H), 7.62 (d, *J* = 7.1 Hz, 1H), 7.56 – 7.49 (m, 2H), 7.03 (t, *J* = 6.3 Hz, 1H), 4.51 (d, *J* = 6.3 Hz, 2H), 4.08 (br.s, 4H), 3.64 (t, *J* = 4.8 Hz, 4H).

^{13}C NMR (101 MHz, DMSO-*d*$_6$) δ 158.5 (C), 153.8 (C), 153.4 (C), 143.1 (C), 135.1 (CH), 131.1 (CH), 129.1 (CH), 128.9 (C), 128.6 (C), 123.5 (CH, d, *J* = 3.9 Hz), 123.0 (CH, d, *J* = 3.7 Hz), 113.4 (C), 66.2 (CH_2), 44.9 (CH_2), 44.2 (CH_2).

HPLC: t_R = 4.63 min, > 98% purity (214 and 254 nm).

HRMS: (ESI+) calc. m/z for $[C_{17}H_{17}F_3N_6O + H]^+$ 379.1489, found 379.1499.

***N*^{6}-(Tetrahydro-2*H*-pyran-4-yl)-*N*^{2}-(3-(trifluoromethyl)benzyl)-9*H*-purine-2,6-diamine (42)**

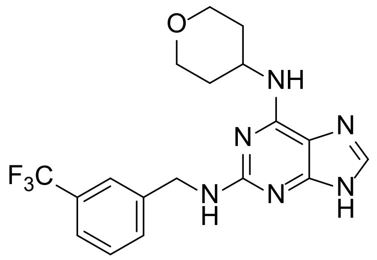

General procedure IV from 5a – The concentrated residue was purified by column chromatography (1% 7 N NH_3 in methanol, 2% methanol and 97% dichloromethane) to give the title compound as a yellow powder (45 mg, 75% yield).

TLC: *Rf* = 0.27 (1% 7 N NH_3 in methanol, 2% methanol and 97% dichloromethane).

^{1}H NMR (401 MHz, DMSO-*d*$_6$) δ 12.15 (br.s, 1H), 7.64 – 7.60 (m, 3H), 7.54 – 7.49 (m, 2H), 7.04 – 6.95 (m, 2H), 4.51 (d, J = 6.3 Hz, 2H), 4.11 – 4.09 (m, 1H), 3.83 (d, J = 11.5 Hz, 2H), 3.30 – 3.27 (m, 2H), 1.69 – 1.54 (m, 4H).

^{13}C NMR (101 MHz, DMSO-*d*$_6$) δ 159.1 (C), 143.4 (C), 135.2 (CH), 131.0 (CH), 129.0 (CH), 128.8 (C, q, J = 31.3 Hz), 125.8 (C), 123.2 (CH, d, J = 3.9 Hz), 123.1 (C), 122.9 (CH, d, J = 3.9 Hz), 66.4 (CH_2), 44.3 (CH_2), 32.6 (CH_2). Two quaternary carbons and one aliphatic CH not observed.

HPLC: t_R = 4.59 min, > 98% purity (214 and 254 nm).

HRMS: (ESI+) calc. m/z for $[C_{18}H_{19}F_3N_6O + H]^+$ 393.1645, found 393.1654.

N^6-(Pyridin-3-ylmethyl)-*N*^{2}-(3-(trifluoromethyl)benzyl)-9*H*-purine-2,6-diamine (43) [35]

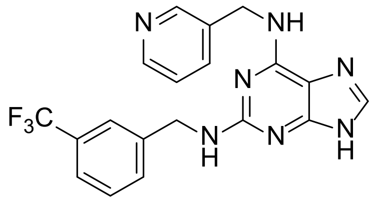

General procedure IV from 5a – The concentrated residue was purified by column chromatography (1% 7 N NH_3 in methanol, 4% methanol and 95% dichloromethane) to give the title compound as a white powder (28 mg, 46% yield).

TLC: *Rf* = 0.41 (1% 7 N NH_3 in methanol, 4% methanol and 95% dichloromethane).

^{1}H NMR (401 MHz, DMSO-*d*$_6$) δ 8.54 (s, 1H), 8.40 (dd, J = 4.8, 1.7 Hz, 1H), 7.85 (br.s, 1H), 7.67 – 7.46 (m, 7H), 7.24 (br. t, J = 6.4 Hz, 1H), 6.99 (br.s, 1H), 4.59 – 4.50 (m, 4H).

^{13}C NMR (101 MHz, DMSO-*d*$_6$) δ 159.1 (C), 149.0 (CH), 147.8 (CH), 143.2 (C), 135.2 (CH), 131.1 (CH), 129.1 (CH), 129.0 (C), 123.3 (CH), 123.3 (d, J = 3.9 Hz, CH), 123.0 (d, J = 3.5 Hz, CH), 44.1 (CH_2). Four quaternary carbons, one aromatic CH and one aliphatic CH_2 not observed.

HPLC: t_R = 3.76 min, > 97% purity (214 and 254 nm).

HRMS: (ESI+) calc. m/z for $[C_{19}H_{16}F_3N_7 + H]^+$ 400.1492, found: 400.1502.

N^6-(4-Methoxybenzyl)-*N*^{2}-(3-(trifluoromethyl)benzyl)-9*H*-purine-2,6-diamine (44)

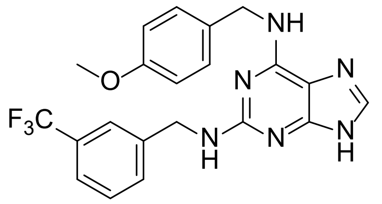

General procedure IV from 5a – The concentrated residue was added cold dichloromethane. The precipitate was collected and was then washed with 0.5 M HCl followed by 30% ammonia solution. The title compound was collected as a beige powder (63 mg, 97% yield).

TLC: *Rf* = 0.43 (1% 7 N NH_3 in methanol, 5% methanol and 94% dichloromethane).

^{1}H NMR (401 MHz, DMSO-*d*$_6$) δ 12.14 (br.s, 1H), 7.64 (s, 2H), 7.59 – 7.47 (m, 4H), 7.21 (d, *J* = 8.2 Hz, 2H), 6.95 (s, 1H), 6.77 (d, *J* = 8.1 Hz, 2H), 4.53 (d, *J* = 6.4 Hz, 4H), 3.69 (s, 3H).

^{13}C NMR (101 MHz, DMSO-*d*$_6$) δ 159.2 (C), 158.0 (C), 143.3 (C), 135.4 (CH), 132.6 (C), 131.2 (CH), 129.1 (CH), 129.0 (C), 128.8 (CH), 125.8 (C), 123.4 (d, J = 3.5 Hz, CH), 123.1 (C), 123.0 (d, J = 3.9 Hz, CH), 113.5 (CH), 55.0 (CH3), 44.2 (CH2). Two quaternary carbons not observed.

HPLC: t_R = 5.33 min, > 95% purity (214 and 254 nm).

HRMS: (ESI+) calc. m/z for $[C_{21}H_{19}F_3N_6O + H]^+$ 429.1645, found: 429.1645.

N^6-Benzyl-9-isopropyl-*N*^{2}-(3-(trifluoromethyl)benzyl)-9*H*-purine-2,6-diamine (45)

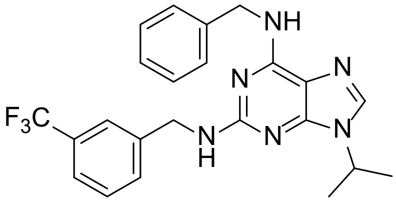

To a solution of *N*^{6}-benzyl-*N*^{2}-(3-(trifluoromethyl)benzyl)-9*H*-purine-2,6-diamine (**9**, 30 mg, 75 µmol) in dimethyl sulfoxide (3 mL), was added 2-bromopropane (23 mg, 18 µL, 188 µmol) and potassium carbonate (31 mg, 226 µmol). The reaction was heated to 80 °C and stirred overnight (16 hours). To the mixture was added water (5 mL) and the precipitate that formed was collected. The title compound was a white powder (28 mg, 85% yield).

TLC: *Rf* = 0.55 (5% methanol in dichloromethane).

^{1}H NMR (401 MHz, DMSO-*d*$_6$) δ 7.78 – 7.78 (m, 2H), 7.68 (s, 1H), 7.59 (d, *J* = 7.3 Hz, 1H), 7.53 (d, *J* = 7.6 Hz, 1H), 7.47 (t, *J* = 7.7 Hz, 1H), 7.26 – 7.15 (m, 5H), 7.09 – 7.06 (m, 1H), 4.57 – 4.47 (m, 5H), 1.42 (d, *J* = 6.8 Hz, 6H).

^{13}C NMR (101 MHz, DMSO-*d*$_6$) δ 158.7 (C), 143.2 (C), 140.6 (C), 135.3 (CH), 131.5 (CH), 128.9 (CH), 128.5 (C), 128.0 (CH), 127.3 (CH), 126.4 (CH), 123.0 (CH), 45.8 (CH), 44.3 (CH_2), 21.9 (CH_3). Four quaternary carbons and one aromatic CH not observed.

HPLC: t_R = 5.90 min, > 95% purity (214 and 254 nm).

HRMS: (ESI+) calc. m/z for $[C_{23}H_{23}F_3N_6 + H]^+$ 441.2009, found 441.2021.

***N*²-(3-Bromobenzyl)-*N*⁶-(2-methoxyethyl)-9-methyl-9*H*-purine-2,6-diamine (46)**

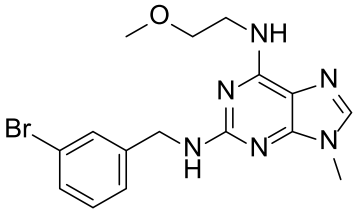

To a solution of N^2-(3-bromobenzyl)-N^6-(2-methoxyethyl)-9*H*-purine-2,6-diamine (**31**, 30 mg, 80 µmol) in anhydrous *N*,*N*-dimethylformamide (3 mL), was added iodomethane (12 mg, 5.5 µL, 87 µmol) and potassium carbonate (12 mg, 87 µmol). The reaction was stirred at room temperature for 8 hours. To the mixture was added water (15 mL) and extracted with ethyl acetate (3 x 15 mL). The organic extracts were combined, washed with water (50 mL) and brine (50 mL), dried over $MgSO_4$ and concentrated *in vacuo*. The residue was purified by column chromatography (3% methanol in dichloromethane) to give the title compound as a white powder (21 mg, 68% yield).

TLC: *Rf* = 0.30 (3% methanol in dichloromethane).

^{1}H NMR (401 MHz, DMSO-*d*$_6$) δ 7.66 (s, 1H), 7.52 (s, 1H), 7.38 – 7.32 (m, 2H), 7.24 (t, *J* = 7.7 Hz, 1H), 7.11 – 7.05 (m, 2H), 4.44 (d, *J* = 6.4 Hz, 2H), 3.54 – 3.51 (m, 5H), 3.40 – 3.38 (m, 2H), 3.20 (s, 3H).

^{13}C NMR (101 MHz, DMSO-*d*$_6$) δ 159.0 (C), 154.5 (C), 144.7 (C), 137.9 (CH), 130.2 (CH), 130.0 (CH), 129.0 (CH), 126.3 (CH), 121.4 (C), 70.5 (CH_2), 57.8 (CH_3), 44.1 (CH_2), 28.9 (CH_3).

HPLC: t_R = 4.64 min, > 95% purity (214 and 254 nm).

HRMS: (ESI+) calc. m/z for $[C_{16}H_{19}{}^{79}BrN_6O + H]^+$ 391.0876, found 391.0887.

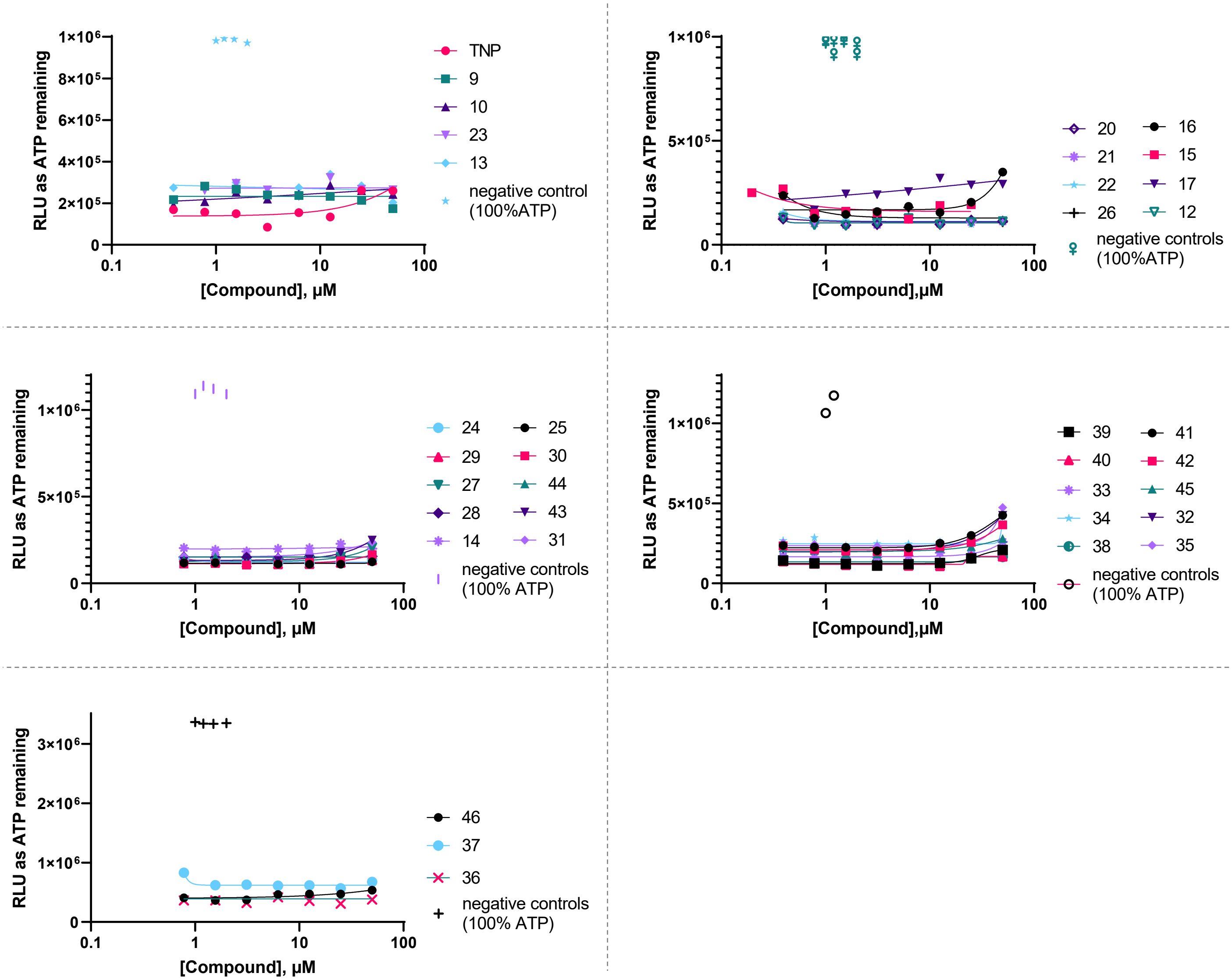

Figure S1. TNP and its analogues do not inhibit *Ca*Ipk2 at concentrations up to 50µM. The IC_{50} for **11**, **18** and **19** were not determined due to poor solubility. IC_{50} values were obtained using the enzyme assay method outlined in section 2.6.

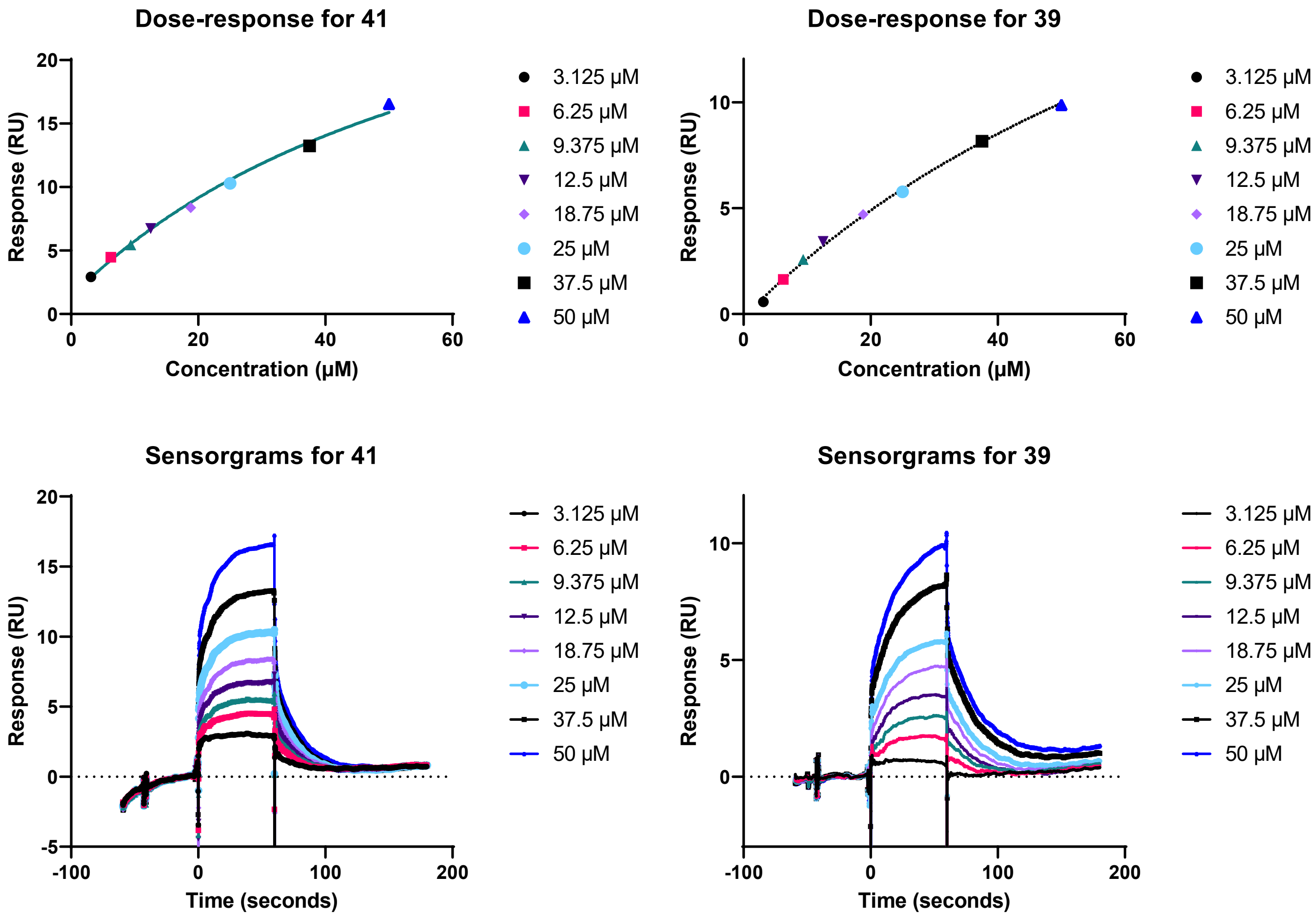

Figure S2. Surface plasmon resonance (SPR) sensorgrams and dose-response curves of compounds **39** and **41** are shown as representative data. The K_D values in **Table 7** were obtained using steady-state affinity.
